# 3β-Acet­oxy-19-hy­droxy-Δ^5^-pregnen-20-one

**DOI:** 10.1107/S1600536813002493

**Published:** 2013-01-31

**Authors:** Aike Meier zu Greffen, Darius P. Kranz, Jörg-M. Neudörfl, Hans-Günther Schmalz

**Affiliations:** a Department für Chemie der Universität zu Köln, Greinstrasse 4, 50939 Köln, Germany

## Abstract

In the title compound, C_23_H_34_O_4_, the *C*/*D* and *D*/*E* rings are *trans* fused and the *A*/*B* ring possesses an *anti* fusion. The two cyclo­hexane rings adopt a chair conformation while the cyclo­hexene ring exhibits a half-chair conformation. The cyclo­pentane ring displays an envelope conformation with the C atom bearing the methyl group as the flap. In the crystal, the mol­ecules are linked by O—H⋯O hydrogen bonds, forming chains along the *b* axis.

## Related literature

For an overview of steroids as biologically important mol­ecules, see: Fieser & Fieser (1961) ▶; Hanson (2010 ▶). For examples of steroids possessing a rearranged *A*/*B*-ring system, see: Du *et al.* (2008 ▶); Aoki *et al.* (2006 ▶); Flyer *et al.* (2010 ▶). For related C-19-functionalized steroids, see: El Sheikh *et al.* (2007 ▶); Shenvi *et al.* (2008 ▶). For an overview of remote functionalization, see: Reese (2001 ▶); Heusler & Kalvoda (1964 ▶). For the first synthesis of the title compound, see: Halpern *et al.* (1963 ▶). For examples of the title compound as an inter­mediate for rearranged *A*/*B*-ring systems, see: Knox *et al.* (1965 ▶); Kranz *et al.* (2011 ▶). For a description of the Cambridge Structural Database, see: Allen (2002 ▶).
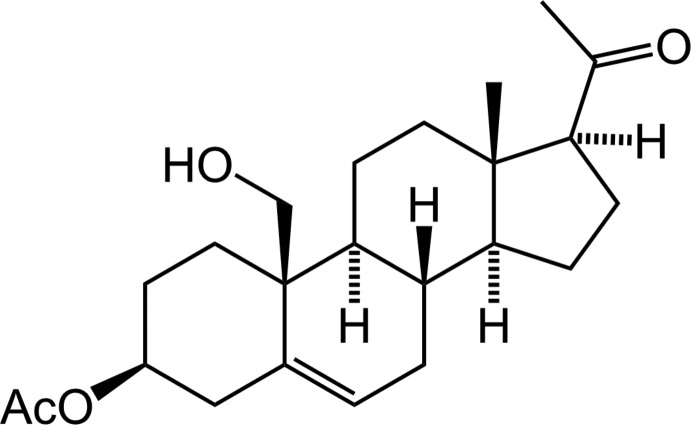


## Experimental

### 

#### Crystal data

C_23_H_34_O_4_
*M* *_r_* = 374.50
Orthorhombic, 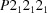

*a* = 8.6960 (6) Å
*b* = 12.3708 (4) Å
*c* = 18.3303 (10) Å
*V* = 1971.91 (18) Å^3^
*Z* = 4
Mo *K*α radiation
μ = 0.08 mm^−1^
*T* = 100 K
0.3 × 0.3 × 0.3 mm

#### Data collection

Nonius KappaCCD diffractometer
9889 measured reflections
2457 independent reflections
1943 reflections with *I* > 2σ(*I*)
*R*_int_ = 0.044

#### Refinement

*R*[*F* ^2^ > 2σ(*F* ^2^)] = 0.037
*wR*(*F* ^2^) = 0.079
*S* = 0.99
2457 reflections
247 parameters
H-atom parameters constrained
Δρ_max_ = 0.23 e Å^−3^
Δρ_min_ = −0.23 e Å^−3^

### 

Data collection: *COLLECT* (Hooft 1998 ▶); cell refinement: *DENZO* (Otwinowski & Minor, 1997 ▶); data reduction: *DENZO*; program(s) used to solve structure: *SHELXS97* (Sheldrick, 2008 ▶); program(s) used to refine structure: *SHELXL97* (Sheldrick, 2008 ▶); molecular graphics: *SCHAKAL99* (Keller, 1999 ▶); software used to prepare material for publication: *PLATON* (Spek, 2009 ▶).

## Supplementary Material

Crystal structure: contains datablock(s) global, I. DOI: 10.1107/S1600536813002493/lr2076sup1.cif

Structure factors: contains datablock(s) I. DOI: 10.1107/S1600536813002493/lr2076Isup2.hkl

Additional supplementary materials:  crystallographic information; 3D view; checkCIF report

## Figures and Tables

**Table 1:** Hydrogen-bond geometry (Å, °)

*D*—H⋯*A*	*D*—H	H⋯*A*	*D*⋯*A*	*D*—H⋯*A*
O3—H3*A*⋯O2^i^	0.84	2.19	2.9305 (19)	147

Symmetry code: (i) 
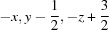
.

**Fig. 1.**
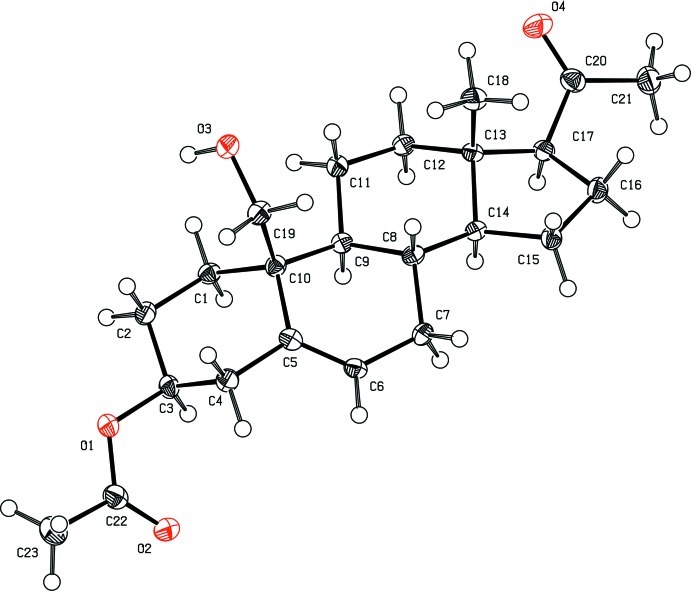
Molecular structure of the title compound. Displacement ellipsoids are drawn at the 50% probability level.

**Fig. 2.**
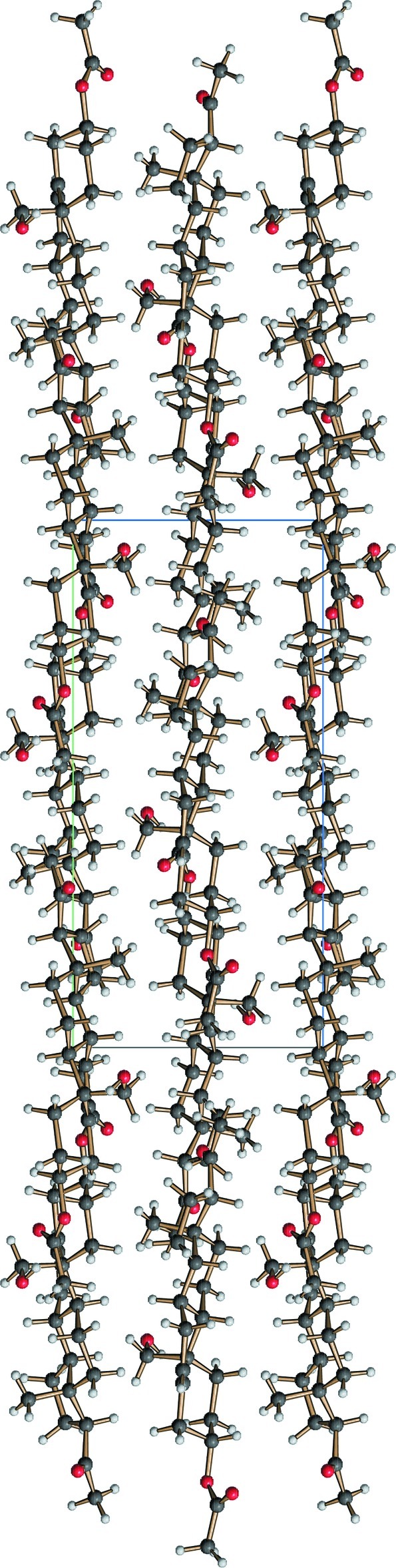
Plot of the unit cell; the b axis is perpendicular to the plane of the paper and the a and c axes are horizontal and vertical, respectively.

## supplementary crystallographic information

### Comment

The structural diversity of steroids as well as their unsurpassed biological potential qualify them as challenging targets for chemical synthesis and as lead structures for pharmacological research (Fieser & Fieser, 1961; Hanson, 2010). In recent years, some unusual steroids displaying a rearranged A/B-ring system with promising biological properties have been reported (Du *et al.*, 2008; Aoki *et al.*, 2006; Flyer *et al.*, 2010). Important intermediates for the synthesis of such rearranged derivatives are C-19 functionalized steroids (El Sheikh *et al.*, 2007; Shenvi *et al.*, 2008). The functionalization of the unactivated angular C-10 methyl group is achieved by remote functionalization (Heusler *et al.*, 1964; Reese, 2001). During our synthesis of diverse B-homo-steroids the *C*-19-hydroxy-steroid (I) was isolated as an intermediate (Kranz *et al.*, 2011). The C/D and D/E rings in C_23_H_34_O_4_ are *trans* fused and the A/B ring possesses an anti fusion. The A ring and the C ring adopt a chair conformation, while the other six membered B ring displays a half chair conformation (Fig. 1). In the five membered ring, the atoms show an envelope conformation and C14/C15/C16/C17 are nearly coplanar while the C13 deviates from the plane by 0.726 (2) Å. The C(5)–C(10)–C(19)–O(3) torsion angle is -165.71 (16)° and the torsion angles C(4)–C(5)–C(10)–C(1) with -45.0 (2)°, C(1)–C(10)–C(9)–C(11) with 68.3 (2)° and C(8)–C(14)–C(13)–C(12) with -58.9 (2)° are within the average range (Allen, 2002). The molecules are linked by O–H···O hydrogen bonds between the C19 hydroxy group of one steroid to the ester carbonyl oxygen of the next steroid, forming chain networks along the *b* axis. These chain networks generate layer structures parallel to the *c* axis (Fig. 2).

### Experimental

The title compound C_23_H_34_O_4_ was prepared in 3 steps starting from commercial pregnenolone-acetate (Kranz *et al.*, 2011).

### Refinement

All hydrogen atoms were placed in geometrically idealized positions and refined with using riding model with C—H = 0.95 Å and *U*_iso_(H) = 1.2*U*_eq_(C) for CH, C—H = 0.99 Å and *U*_iso_(H) = 1.2*U*_eq_(C) for CH_2_, C—H = 0.98 Å and *U*_iso_(H) = 1.5*U*_eq_(C) for CH_3 _and OH.

### Crystal data

**Table:**

C_23_H_34_O_4_	*F*(000) = 816
*M**_r_* = 374.50	*D*_x_ = 1.261 Mg m^−^^3^
Orthorhombic, *P*2_1_2_1_2_1_	Mo *K*α radiation, λ = 0.71073 Å
Hall symbol: P 2ac 2ab	Cell parameters from 9889 reflections
*a* = 8.6960 (6) Å	θ = 2.0–27.0°
*b* = 12.3708 (4) Å	µ = 0.08 mm^−^^1^
*c* = 18.3303 (10) Å	*T* = 100 K
*V* = 1971.91 (18) Å^3^	Prism, colourless
*Z* = 4	0.3 × 0.3 × 0.3 mm

### Data collection

**Table:**

Nonius KappaCCD diffractometer	1943 reflections with *I* > 2σ(*I*)
Radiation source: fine-focus sealed tube	*R*_int_ = 0.044
Graphite monochromator	θ_max_ = 27.0°, θ_min_ = 2.0°
Phi/ω–Scans scans	*h* = −6→11
9889 measured reflections	*k* = −12→15
2457 independent reflections	*l* = −21→23

### Refinement

**Table:**

Refinement on *F*^2^	Primary atom site location: structure-invariant direct methods
Least-squares matrix: full	Secondary atom site location: difference Fourier map
*R*[*F*^2^ > 2σ(*F*^2^)] = 0.037	Hydrogen site location: inferred from neighbouring sites
*wR*(*F*^2^) = 0.079	H-atom parameters constrained
*S* = 0.99	*w* = 1/[σ^2^(*F*_o_^2^) + (0.0425*P*)^2^] where *P* = (*F*_o_^2^ + 2*F*_c_^2^)/3
2457 reflections	(Δ/σ)_max_ < 0.001
247 parameters	Δρ_max_ = 0.23 e Å^−^^3^
0 restraints	Δρ_min_ = −0.23 e Å^−^^3^

### Special details

**Table:**

Geometry. All e.s.d.'s (except the e.s.d. in the dihedral angle between two l.s. planes) are estimated using the full covariance matrix. The cell e.s.d.'s are taken into account individually in the estimation of e.s.d.'s in distances, angles and torsion angles; correlations between e.s.d.'s in cell parameters are only used when they are defined by crystal symmetry. An approximate (isotropic) treatment of cell e.s.d.'s is used for estimating e.s.d.'s involving l.s. planes.
Refinement. Refinement of *F*^2^ against ALL reflections. The weighted *R*-factor *wR* and goodness of fit *S* are based on *F*^2^, conventional *R*-factors *R* are based on *F*, with *F* set to zero for negative *F*^2^. The threshold expression of *F*^2^ > σ(*F*^2^) is used only for calculating *R*-factors(gt) *etc*. and is not relevant to the choice of reflections for refinement. *R*-factors based on *F*^2^ are statistically about twice as large as those based on *F*, and *R*- factors based on ALL data will be even larger.

### Fractional atomic coordinates and isotropic or equivalent isotropic displacement parameters (Å^2^)

**Table:**

	*x*	*y*	*z*	*U*_iso_*/*U*_eq_	
O1	−0.03545 (17)	0.54114 (10)	0.67613 (8)	0.0195 (3)	
O2	−0.13591 (19)	0.70584 (11)	0.65410 (8)	0.0267 (4)	
O3	0.20344 (19)	0.38623 (10)	0.94444 (8)	0.0246 (4)	
H3A	0.1481	0.3467	0.9180	0.037*	
O4	−0.0183 (2)	0.54920 (11)	1.30301 (9)	0.0360 (5)	
C1	−0.0748 (2)	0.47261 (15)	0.87606 (11)	0.0170 (5)	
H1A	−0.0708	0.4021	0.9016	0.020*	
H1B	−0.1789	0.5030	0.8836	0.020*	
C2	−0.0516 (3)	0.45275 (14)	0.79398 (11)	0.0182 (5)	
H2A	0.0481	0.4161	0.7856	0.022*	
H2B	−0.1345	0.4055	0.7752	0.022*	
C3	−0.0541 (3)	0.55950 (14)	0.75430 (11)	0.0180 (5)	
H3	−0.1547	0.5964	0.7634	0.022*	
C4	0.0760 (3)	0.63061 (15)	0.78170 (11)	0.0188 (5)	
H4A	0.1758	0.5953	0.7713	0.023*	
H4B	0.0738	0.7007	0.7557	0.023*	
C5	0.0611 (2)	0.64986 (15)	0.86293 (11)	0.0165 (5)	
C6	0.0610 (3)	0.75048 (15)	0.88802 (11)	0.0172 (5)	
H6	0.0682	0.8072	0.8532	0.021*	
C7	0.0504 (3)	0.78158 (14)	0.96614 (11)	0.0172 (5)	
H7A	−0.0510	0.8155	0.9751	0.021*	
H7B	0.1306	0.8360	0.9769	0.021*	
C8	0.0700 (3)	0.68595 (14)	1.01773 (11)	0.0160 (5)	
H8	0.1819	0.6683	1.0216	0.019*	
C9	−0.0163 (2)	0.58588 (14)	0.98836 (11)	0.0165 (5)	
H9	−0.1252	0.6093	0.9807	0.020*	
C10	0.0442 (2)	0.54967 (14)	0.91196 (11)	0.0161 (5)	
C11	−0.0222 (3)	0.49458 (14)	1.04593 (11)	0.0193 (5)	
H20A	−0.0894	0.4359	1.0277	0.023*	
H20B	0.0825	0.4644	1.0522	0.023*	
C12	−0.0822 (3)	0.53265 (15)	1.12077 (11)	0.0185 (5)	
H12A	−0.1904	0.5564	1.1159	0.022*	
H12B	−0.0793	0.4716	1.1556	0.022*	
C13	0.0157 (2)	0.62660 (15)	1.15054 (11)	0.0158 (5)	
C14	0.0106 (3)	0.71672 (14)	1.09252 (11)	0.0154 (5)	
H14	−0.1005	0.7351	1.0859	0.018*	
C15	0.0832 (3)	0.81413 (15)	1.13106 (11)	0.0191 (5)	
H15A	0.0457	0.8828	1.1098	0.023*	
H15B	0.1967	0.8119	1.1274	0.023*	
C16	0.0306 (3)	0.80233 (14)	1.21114 (11)	0.0216 (5)	
H16A	−0.0408	0.8614	1.2246	0.026*	
H16B	0.1201	0.8042	1.2445	0.026*	
C17	−0.0521 (3)	0.69056 (14)	1.21538 (11)	0.0173 (5)	
H17	−0.1627	0.7040	1.2035	0.021*	
C18	0.1795 (3)	0.58958 (16)	1.16724 (12)	0.0232 (5)	
H18A	0.1764	0.5277	1.2006	0.035*	
H18B	0.2366	0.6489	1.1900	0.035*	
H18C	0.2306	0.5683	1.1218	0.035*	
C19	0.2043 (2)	0.49419 (15)	0.91585 (12)	0.0199 (5)	
H19A	0.2488	0.4924	0.8661	0.024*	
H19B	0.2728	0.5391	0.9466	0.024*	
C20	−0.0486 (3)	0.64326 (16)	1.29112 (11)	0.0219 (5)	
C21	−0.0887 (3)	0.71767 (17)	1.35354 (11)	0.0293 (6)	
H21A	0.0035	0.7569	1.3691	0.044*	
H21B	−0.1286	0.6750	1.3944	0.044*	
H21C	−0.1672	0.7694	1.3376	0.044*	
C22	−0.0763 (3)	0.62330 (17)	0.63230 (12)	0.0221 (5)	
C23	−0.0378 (3)	0.60069 (18)	0.55382 (12)	0.0315 (6)	
H23A	0.0720	0.6136	0.5456	0.047*	
H23B	−0.0981	0.6485	0.5223	0.047*	
H23C	−0.0622	0.5252	0.5424	0.047*	

### Atomic displacement parameters (Å^2^)

**Table:**

	*U*^11^	*U*^22^	*U*^33^	*U*^12^	*U*^13^	*U*^23^
O1	0.0238 (9)	0.0196 (7)	0.0151 (8)	0.0004 (7)	0.0002 (7)	−0.0013 (6)
O2	0.0370 (10)	0.0199 (8)	0.0232 (9)	0.0036 (7)	−0.0009 (8)	0.0023 (7)
O3	0.0298 (10)	0.0191 (7)	0.0248 (9)	0.0059 (7)	−0.0058 (8)	−0.0034 (6)
O4	0.0604 (13)	0.0203 (8)	0.0274 (10)	0.0038 (9)	0.0007 (9)	0.0066 (6)
C1	0.0157 (11)	0.0153 (10)	0.0200 (11)	−0.0005 (9)	−0.0012 (9)	0.0014 (8)
C2	0.0176 (12)	0.0158 (10)	0.0213 (12)	0.0008 (9)	−0.0014 (10)	−0.0024 (8)
C3	0.0208 (12)	0.0187 (10)	0.0145 (11)	0.0006 (10)	0.0020 (10)	−0.0025 (8)
C4	0.0200 (12)	0.0184 (10)	0.0180 (11)	−0.0006 (9)	0.0025 (10)	0.0005 (9)
C5	0.0117 (11)	0.0207 (11)	0.0171 (12)	−0.0011 (9)	0.0000 (9)	0.0013 (8)
C6	0.0175 (12)	0.0184 (10)	0.0156 (11)	−0.0014 (9)	−0.0013 (10)	0.0030 (8)
C7	0.0180 (12)	0.0136 (10)	0.0199 (11)	−0.0022 (9)	0.0004 (10)	−0.0003 (8)
C8	0.0148 (11)	0.0144 (10)	0.0188 (12)	−0.0007 (9)	0.0010 (10)	0.0018 (8)
C9	0.0179 (12)	0.0151 (10)	0.0166 (11)	−0.0003 (9)	−0.0002 (9)	0.0008 (8)
C10	0.0151 (11)	0.0149 (10)	0.0185 (11)	−0.0005 (9)	−0.0008 (9)	−0.0007 (8)
C11	0.0239 (13)	0.0165 (10)	0.0174 (12)	−0.0011 (9)	−0.0008 (9)	−0.0001 (8)
C12	0.0221 (12)	0.0153 (10)	0.0183 (11)	−0.0025 (9)	−0.0002 (10)	0.0018 (9)
C13	0.0172 (11)	0.0162 (10)	0.0139 (11)	0.0002 (9)	−0.0005 (9)	0.0022 (8)
C14	0.0147 (11)	0.0138 (10)	0.0177 (11)	0.0004 (9)	−0.0003 (9)	0.0011 (8)
C15	0.0226 (12)	0.0167 (10)	0.0179 (12)	−0.0032 (9)	−0.0010 (10)	−0.0011 (8)
C16	0.0283 (14)	0.0161 (10)	0.0204 (12)	−0.0047 (10)	0.0007 (11)	−0.0028 (8)
C17	0.0190 (12)	0.0162 (10)	0.0166 (11)	−0.0017 (9)	−0.0023 (10)	0.0011 (8)
C18	0.0236 (13)	0.0228 (11)	0.0232 (13)	0.0034 (10)	−0.0024 (10)	0.0022 (10)
C19	0.0164 (12)	0.0185 (11)	0.0250 (12)	0.0001 (9)	−0.0017 (10)	−0.0007 (9)
C20	0.0219 (13)	0.0230 (11)	0.0208 (12)	−0.0033 (10)	−0.0033 (11)	0.0022 (9)
C21	0.0440 (17)	0.0237 (12)	0.0203 (13)	−0.0065 (11)	0.0002 (12)	−0.0009 (9)
C22	0.0225 (12)	0.0230 (12)	0.0208 (12)	−0.0050 (10)	−0.0024 (11)	0.0019 (9)
C23	0.0393 (17)	0.0329 (12)	0.0223 (13)	−0.0003 (12)	0.0019 (12)	−0.0001 (10)

### Geometric parameters (Å, º)

**Table:**

O1—C22	1.344 (2)	C11—C12	1.542 (3)
O1—C3	1.460 (2)	C11—H20A	0.9900
O2—C22	1.213 (2)	C11—H20B	0.9900
O3—C19	1.435 (2)	C12—C13	1.541 (3)
O3—H3A	0.8400	C12—H12A	0.9900
O4—C20	1.213 (2)	C12—H12B	0.9900
C1—C2	1.538 (3)	C13—C18	1.527 (3)
C1—C10	1.553 (3)	C13—C14	1.541 (3)
C1—H1A	0.9900	C13—C17	1.545 (3)
C1—H1B	0.9900	C14—C15	1.533 (3)
C2—C3	1.508 (3)	C14—H14	1.0000
C2—H2A	0.9900	C15—C16	1.545 (3)
C2—H2B	0.9900	C15—H15A	0.9900
C3—C4	1.519 (3)	C15—H15B	0.9900
C3—H3	1.0000	C16—C17	1.561 (3)
C4—C5	1.513 (3)	C16—H16A	0.9900
C4—H4A	0.9900	C16—H16B	0.9900
C4—H4B	0.9900	C17—C20	1.507 (3)
C5—C6	1.327 (3)	C17—H17	1.0000
C5—C10	1.538 (3)	C18—H18A	0.9800
C6—C7	1.486 (3)	C18—H18B	0.9800
C6—H6	0.9500	C18—H18C	0.9800
C7—C8	1.524 (2)	C19—H19A	0.9900
C7—H7A	0.9900	C19—H19B	0.9900
C7—H7B	0.9900	C20—C21	1.509 (3)
C8—C14	1.514 (3)	C21—H21A	0.9800
C8—C9	1.544 (3)	C21—H21B	0.9800
C8—H8	1.0000	C21—H21C	0.9800
C9—C11	1.547 (3)	C22—C23	1.503 (3)
C9—C10	1.562 (3)	C23—H23A	0.9800
C9—H9	1.0000	C23—H23B	0.9800
C10—C19	1.554 (3)	C23—H23C	0.9800
			
C22—O1—C3	116.09 (15)	C11—C12—H12A	109.4
C19—O3—H3A	109.5	C13—C12—H12B	109.4
C2—C1—C10	115.17 (17)	C11—C12—H12B	109.4
C2—C1—H1A	108.5	H12A—C12—H12B	108.0
C10—C1—H1A	108.5	C18—C13—C12	111.11 (16)
C2—C1—H1B	108.5	C18—C13—C14	112.45 (17)
C10—C1—H1B	108.5	C12—C13—C14	106.58 (16)
H1A—C1—H1B	107.5	C18—C13—C17	110.81 (18)
C3—C2—C1	109.27 (15)	C12—C13—C17	116.62 (17)
C3—C2—H2A	109.8	C14—C13—C17	98.59 (14)
C1—C2—H2A	109.8	C8—C14—C15	118.33 (17)
C3—C2—H2B	109.8	C8—C14—C13	115.67 (15)
C1—C2—H2B	109.8	C15—C14—C13	103.82 (16)
H2A—C2—H2B	108.3	C8—C14—H14	106.0
O1—C3—C2	109.60 (14)	C15—C14—H14	106.0
O1—C3—C4	109.38 (16)	C13—C14—H14	106.0
C2—C3—C4	109.69 (17)	C14—C15—C16	103.98 (16)
O1—C3—H3	109.4	C14—C15—H15A	111.0
C2—C3—H3	109.4	C16—C15—H15A	111.0
C4—C3—H3	109.4	C14—C15—H15B	111.0
C5—C4—C3	110.67 (17)	C16—C15—H15B	111.0
C5—C4—H4A	109.5	H15A—C15—H15B	109.0
C3—C4—H4A	109.5	C15—C16—C17	105.52 (15)
C5—C4—H4B	109.5	C15—C16—H16A	110.6
C3—C4—H4B	109.5	C17—C16—H16A	110.6
H4A—C4—H4B	108.1	C15—C16—H16B	110.6
C6—C5—C4	119.25 (17)	C17—C16—H16B	110.6
C6—C5—C10	123.59 (18)	H16A—C16—H16B	108.8
C4—C5—C10	117.15 (16)	C20—C17—C13	120.13 (16)
C5—C6—C7	125.24 (18)	C20—C17—C16	112.36 (17)
C5—C6—H6	117.4	C13—C17—C16	103.86 (16)
C7—C6—H6	117.4	C20—C17—H17	106.6
C6—C7—C8	112.96 (16)	C13—C17—H17	106.6
C6—C7—H7A	109.0	C16—C17—H17	106.6
C8—C7—H7A	109.0	C13—C18—H18A	109.5
C6—C7—H7B	109.0	C13—C18—H18B	109.5
C8—C7—H7B	109.0	H18A—C18—H18B	109.5
H7A—C7—H7B	107.8	C13—C18—H18C	109.5
C14—C8—C7	109.19 (15)	H18A—C18—H18C	109.5
C14—C8—C9	110.59 (17)	H18B—C18—H18C	109.5
C7—C8—C9	110.59 (16)	O3—C19—C10	115.04 (17)
C14—C8—H8	108.8	O3—C19—H19A	108.5
C7—C8—H8	108.8	C10—C19—H19A	108.5
C9—C8—H8	108.8	O3—C19—H19B	108.5
C8—C9—C11	111.31 (16)	C10—C19—H19B	108.5
C8—C9—C10	112.25 (16)	H19A—C19—H19B	107.5
C11—C9—C10	114.42 (15)	O4—C20—C17	122.86 (19)
C8—C9—H9	106.1	O4—C20—C21	119.94 (19)
C11—C9—H9	106.1	C17—C20—C21	117.18 (17)
C10—C9—H9	106.1	C20—C21—H21A	109.5
C5—C10—C1	108.10 (16)	C20—C21—H21B	109.5
C5—C10—C19	107.29 (16)	H21A—C21—H21B	109.5
C1—C10—C19	110.20 (15)	C20—C21—H21C	109.5
C5—C10—C9	108.97 (15)	H21A—C21—H21C	109.5
C1—C10—C9	109.34 (17)	H21B—C21—H21C	109.5
C19—C10—C9	112.81 (17)	O2—C22—O1	123.6 (2)
C12—C11—C9	113.28 (15)	O2—C22—C23	124.6 (2)
C12—C11—H20A	108.9	O1—C22—C23	111.88 (18)
C9—C11—H20A	108.9	C22—C23—H23A	109.5
C12—C11—H20B	108.9	C22—C23—H23B	109.5
C9—C11—H20B	108.9	H23A—C23—H23B	109.5
H20A—C11—H20B	107.7	C22—C23—H23C	109.5
C13—C12—C11	111.00 (17)	H23A—C23—H23C	109.5
C13—C12—H12A	109.4	H23B—C23—H23C	109.5
			
C10—C1—C2—C3	−57.1 (2)	C9—C11—C12—C13	−56.8 (2)
C22—O1—C3—C2	162.62 (17)	C11—C12—C13—C18	−65.7 (2)
C22—O1—C3—C4	−77.1 (2)	C11—C12—C13—C14	57.1 (2)
C1—C2—C3—O1	−178.82 (16)	C11—C12—C13—C17	166.01 (16)
C1—C2—C3—C4	61.1 (2)	C7—C8—C14—C15	−58.5 (2)
O1—C3—C4—C5	−179.18 (15)	C9—C8—C14—C15	179.59 (17)
C2—C3—C4—C5	−58.9 (2)	C7—C8—C14—C13	177.42 (17)
C3—C4—C5—C6	−126.4 (2)	C9—C8—C14—C13	55.5 (2)
C3—C4—C5—C10	52.6 (2)	C18—C13—C14—C8	63.1 (2)
C4—C5—C6—C7	−178.7 (2)	C12—C13—C14—C8	−58.9 (2)
C10—C5—C6—C7	2.3 (4)	C17—C13—C14—C8	179.88 (18)
C5—C6—C7—C8	10.9 (3)	C18—C13—C14—C15	−68.3 (2)
C6—C7—C8—C14	−162.88 (18)	C12—C13—C14—C15	169.74 (17)
C6—C7—C8—C9	−41.0 (3)	C17—C13—C14—C15	48.5 (2)
C14—C8—C9—C11	−48.9 (2)	C8—C14—C15—C16	−164.60 (18)
C7—C8—C9—C11	−169.97 (18)	C13—C14—C15—C16	−34.9 (2)
C14—C8—C9—C10	−178.62 (16)	C14—C15—C16—C17	6.9 (2)
C7—C8—C9—C10	60.3 (2)	C18—C13—C17—C20	−51.9 (2)
C6—C5—C10—C1	134.0 (2)	C12—C13—C17—C20	76.5 (2)
C4—C5—C10—C1	−45.0 (2)	C14—C13—C17—C20	−169.98 (19)
C6—C5—C10—C19	−107.2 (2)	C18—C13—C17—C16	74.70 (19)
C4—C5—C10—C19	73.8 (2)	C12—C13—C17—C16	−156.87 (17)
C6—C5—C10—C9	15.2 (3)	C14—C13—C17—C16	−43.38 (19)
C4—C5—C10—C9	−163.72 (18)	C15—C16—C17—C20	154.51 (18)
C2—C1—C10—C5	46.9 (2)	C15—C16—C17—C13	23.2 (2)
C2—C1—C10—C19	−70.0 (2)	C5—C10—C19—O3	−165.71 (16)
C2—C1—C10—C9	165.42 (16)	C1—C10—C19—O3	−48.2 (2)
C8—C9—C10—C5	−45.7 (2)	C9—C10—C19—O3	74.3 (2)
C11—C9—C10—C5	−173.76 (17)	C13—C17—C20—O4	−12.8 (3)
C8—C9—C10—C1	−163.62 (15)	C16—C17—C20—O4	−135.4 (2)
C11—C9—C10—C1	68.3 (2)	C13—C17—C20—C21	168.8 (2)
C8—C9—C10—C19	73.38 (19)	C16—C17—C20—C21	46.2 (3)
C11—C9—C10—C19	−54.7 (2)	C3—O1—C22—O2	−5.2 (3)
C8—C9—C11—C12	51.4 (2)	C3—O1—C22—C23	174.20 (17)
C10—C9—C11—C12	179.96 (18)		

### Hydrogen-bond geometry (Å, º)

**Table:**

*D*—H···*A*	*D*—H	H···*A*	*D*···*A*	*D*—H···*A*
O3—H3*A*···O2^i^	0.84	2.19	2.9305 (19)	147

Symmetry code: (i) −*x*, *y*−1/2, −*z*+3/2.
